# Research and development of novel embolization materials and study on their feasibility of preventing T2EL after EVAR abdominal aortic aneurysm

**DOI:** 10.3389/fcvm.2026.1740816

**Published:** 2026-06-01

**Authors:** E. Erdemutu, Boru Huang, Liqiang Hu, Shaohong Yin, Ying Lan, Chongbin Zhou, Xiangchen Dai

**Affiliations:** 1 Department of Vascular Surgery, Affiliated Hospital of Inner Mongolia Medical University, Huhhot, Inner Mongolia, China; 2 Department of Vascular Surgery, Hohhot First Hospital, Huhhot, Inner Mongolia, China; 3 Department of Vascular Surgery, Tianjin Medical University General Hospital, Tianjin, China

**Keywords:** abdominal aortic aneurysm, prophylactic embolism, thrombin coated coil, type II endoleak, vascular remodeling

## Abstract

**Objective:**

This study aimed to develop a novel vascular embolization material—Thrombin-Coated Controllable Coil (TCC)—and to evaluate its physicochemical properties, biological performance, and feasibility in preventing Type II Endoleak (T2EL) following Endovascular Aneurysm Repair (EVAR) for abdominal aortic aneurysm (AAA).

**Methods:**

TCC was prepared by coating an interlocking detachable coil (IDC) with a polyvinylpyrrolidone/polycaprolactone (PVP/PCL) blend containing lyophilized thrombin powder (LTP). Its morphology, dissolution, and thrombin-loading capacity were characterized. Coagulation efficiency was tested under static and dynamic conditions. The effects of TCC on vascular endothelial (VECs) and smooth muscle cells (VSMCs) were assessed *in vitro*, and a Bama minipig AAA model with T2EL was used to evaluate *in vivo* performance. Transcriptomic analysis was conducted to explore underlying molecular mechanisms.

**Results:**

TCC exhibited a uniform thrombin coating layer, with an average drug loading of 0.0066 ± 0.0003 g, and the water solubility time was 1,080 ± 42.43 s. In the static and dynamic coagulation experiments, compared with the IDC group and the control group, TCC significantly shortened the coagulation time, and its coagulation efficiency was comparable to that of LTP. Extracellularly, TCC promoted apoptosis of VSMCs, inhibited the proliferation and migration of VSMCs, and had a relatively minor effect on VECs. *In vivo*, TCC effectively prevented T2EL, achieved complete thrombosis within the aneurysm, and no complications occurred. Transcriptomic analysis revealed 161 differentially expressed genes, including downregulation of MMP9, VDR, E2F8, RUNX2, and MKI67, and upregulation of BTG2, IGFBP6, LGR5 and DPT.

**Conclusion:**

TCC exhibits excellent biocompatibility, controllability, strong thrombogenic activity and safety. It is a highly promising next-generation embolic material for treating EVAR-related complications.

## 1 Introduction

Abdominal aortic aneurysm (AAA) is a life-threatening vascular disease characterized by localized dilatation of the abdominal aorta exceeding 50% of its normal diameter (1–3). At present, Endovascular Aneurysm Repair (EVAR) treatment is still the preferred treatment for AAA, but anatomical abnormalities account for 30%–50% of AAA patients (4). However, complications such as endoleak, particularly Type II Endoleak (T2EL) caused by retrograde flow from branch arteries, remain a significant clinical challenge. The reported incidence of T2EL varies widely across studies, ranging from approximately 10%–44% depending on patient selection, imaging protocols, and follow-up duration (5, 6). A systematic review reported a mean incidence of 34% (7), while large registry data from the EUROSTAR collaboration showed an isolated T2EL incidence of 9% at three-year follow-up (8). Importantly, the natural history of T2EL exhibits distinct temporal patterns. The early post-EVAR incidence (within 30 days) ranges from 6% to 17%, which decreases to 1%–5% at one year due to spontaneous thrombosis (9). The intermediate-term spontaneous remission situation, that is, the situation where there is an intradural leakage as shown by the initial CT examination but the subsequent CT examination results are negative, has a relatively high incidence. Approximately 33% of patients experienced type II intradural leakage remission during the period of 1–6 months (10). Persistent T2EL beyond six months is associated with aneurysm sac enlargement in 20%–80% of cases (7, 11), and in patients with T2EL, approximately 8.4% experienced cyst dilation exceeding 5 millimeters during a four-year follow-up period (12). Notably, many early T2ELs resolve spontaneously, whereas persistent or late-onset T2ELs are more likely to lead to aneurysm sac enlargement and secondary rupture, with re-intervention required when sac expansion exceeds 10 mm (13, 14), which is closely associated with an increased risk of reoperation (15). Given these differences, the clinical significance of T2EL lies not only in its overall occurrence but also in its temporal characteristics. Identifying effective intraoperative strategies to reduce clinically relevant T2EL remains an important objective in EVAR procedures. Prophylactic embolization of the aneurysm sac or its side branches during EVAR has been proposed as a superior strategy to treating established T2EL (16, 17).

The current embolic materials include standard detachable coils, fibrous or hydrogel-coated coils, as well as glues (such as Glubran, Onix) (18, 19) and other liquid embolic agents. These materials have been used in the treatment of T2EL for many years. However, liquid embolic agents also have inherent limitations: their fluidity can lead to non-targeted embolization (such as mesenteric ischemia, spinal cord ischemia, etc., serious complications), and the intraoperative controllability is poor, requiring extremely high skill from the surgeon (20). To enhance the thrombogenic ability of bare coils, the design of the devices has undergone significant improvements. The fiber-coated coils increase the attachment area of thrombus by wrapping Dacron fibers on the metal wire; while the new generation of hydrogel coils (such as the AZUR series) are coated with cross-linked polymers that expand upon contact with water, significantly improving the filling density and mechanically sealing the blood flow, thereby promoting more stable thrombosis (21, 22). Although the thrombogenic effects of these modified coils are superior to bare coils, each method has specific limitations, including insufficient controllability, non-targeted embolization or low local thrombosis formation efficiency. Thrombin is a potent coagulant capable of rapid clot formation but lacks localized control and poses risks of ectopic embolization. Conversely, interlocking detachable coils (IDC) provide mechanical control but insufficient procoagulant activity. Developing a material that combines the controllability of IDC with the high coagulative efficiency of thrombin remains an unmet challenge in endovascular therapy.

To address this gap, we developed a Thrombin-Coated Controllable Coil (TCC). This system leverages the established delivery and mechanical scaffolding of a fibered IDC coil, augmented with a coating of lyophilized thrombin (LTP) to induce rapid and robust thrombosis. A biodegradable polymer blend of PVP and PCL was used to control the thrombin release profile, ensuring activation occurs at the target site. This study details the development, *in vitro* and *in vivo* evaluation, and preliminary investigation into the molecular mechanisms of the TCC, demonstrating its feasibility as a novel embolic agent for T2EL prevention.

The primary objective of this study was to evaluate whether TCC improves aneurysmal sac thrombosis and prevents T2EL compared with conventional IDC. Secondary analyses were performed to explore potential biological effects on vascular cells and molecular remodeling.

## 2 Materials and methods

### 2.1 Preparation of the TCC

PVP (Shanghai Macklin Biochemical Technology Co., Ltd.) and PCL (Shanghai Macklin Biochemical Technology Co., Ltd.) were dissolved in dichloromethane (Tianjin Zhiyuan Chemical Reagent Co., Ltd.) to form a homogeneous coating medium. The mixture was stirred magnetically at 600 rpm for 10 min at room temperature. An IDC (60 mm; Boston Scientific, USA) was immersed in the medium for 30 min, enabling uniform gel adhesion. The coil was then rolled in LTP (Shanghai Yuanye Bio-Technology Co., Ltd.) and gently compressed to ensure tight adherence. After complete solvent evaporation, a uniform thrombin coating was formed, producing the final TCC construct.

### 2.2 Determination of drug loading and water dissolution time

The thrombin mass difference before and after coating was measured to determine the loading capacity per coil. To evaluate dissolution control, TCCs were immersed in 0.9% sodium chloride solution, and the dissolution time was recorded from immersion until complete coating detachment. Ratios of PVP:PCL were adjusted to optimize water-dissolution duration. The optimal ratio (1.1:0.1 g) achieved an average dissolution time of 1,080 ± 42.4 s.

### 2.3 Coagulation efficiency assays

Static and dynamic coagulation assays were performed using fresh anticoagulated minipig blood. The time from calcium activation to clot formation was recorded. Four groups were compared: TCC, free LTP, IDC, and control. The optical density (OD) of plasma supernatants was monitored over time to assess residual red blood cells, indirectly reflecting coagulation efficiency.

### 2.4 Cell source and culture conditions

Human aortic vascular smooth muscle cells (VSMCs) and human aortic endothelial cells (VECs) were obtained from Wuhan Procell Life Science & Technology Co., Ltd. All cells were primary cells and used between passages 3–8. Cells were cultured in recommended complete medium under standard conditions (37 °C, 5% CO_2_).

For the material exposure experiment, the sterilized TCC or IDC fragments were directly placed in the Transwell chamber to prevent direct cell-to-cell contact. For the LTP group, the free thrombin was dissolved in the culture medium, with its concentration being equal to the mass of thrombin loaded by one TCC, to ensure dose matching.

### 2.5 Cell viability assay

To evaluate the impact of TCC, LTP, and IDC modulation on VECs and VSMCs cell viability, CCK-8 assays were conducted under optimized experimental conditions. After the cells were treated with TCC, LTP and IDC, 10 μL of CCK-8 (CK04, Dojindo Laboratories, Japan) was applied and optical density was recorded at 450 nm. All experiments were conducted using three-well plates and were independently repeated three times.

### 2.6 Cell apoptosis

Vascular smooth muscle cells (VSMCs) and vascular endothelial cells (VECs) were seeded in 6-well plates and treated according to experimental grouping. After treatment, both adherent and floating cells were collected, washed once with phosphate-buffered saline (PBS), and centrifuged at 300–400 g for 5 min at 4 °C. Process the cells according to the instructions of the Annexin V-FITC/PI Apoptosis Detection Kit (Beyotime, Shanghai, China; C1062M). Samples were analyzed using a flow cytometer equipped with a 488 nm laser (CytoFLEX S, mybeckman, USA). Data were analyzed using FlowJo software (Tree Star, USA). All experiments were repeated three times.

### 2.7 Migration experiment

Cell migration was assessed using a Transwell chamber assay. Briefly, vascular smooth muscle cells (VSMCs) or vascular endothelial cells (VECs) were suspended in serum-free medium and seeded into the upper chamber of a 24-well Transwell insert (8-um pore size). The lower chamber was filled with complete medium containing 10% fetal bovine serum (Gibco, USA; 10099141) as a chemoattractant. After incubation for 24 h at 37 °C, non-migrated cells on the upper surface of the membrane were carefully removed with a cotton swab. Cells that had migrated to the lower surface were fixed with 4% paraformaldehyde, stained with 1% crystal violet (Solarbio, Beijing, China; G1062) and imaged under an inverted microscope. The number of migrated cells was counted in five random fields per well using ImageJ software, and the average was calculated. All experiments were repeated three times.

### 2.8 Immunofluorescence

Cells and tissues were fixed with 4% paraformaldehyde (Beyotime, Shanghai, China; P0099) for 15 min and permeabilized with 0.1% Triton X-100 for 10 min at room temperature. After blocking with 5% bovine serum albumin (BSA) (Solarbio, Beijing, China; SW3015) for 1 h, the cells were incubated overnight at 4 °C with a primary antibody against the target protein (e.g., α-SMA). Following washes, the cells were incubated with a fluorophore-conjugated secondary antibody for 1 h at room temperature in the dark. Nuclei were counterstained with DAPI (4′,6-diamidino-2-phenylindole). Fluorescence images were captured using a fluorescence microscope, and the relative fluorescence intensity was quantified using ImageJ software.

### 2.9 *In vivo* animal study

A total of six 32-week-old Bama miniature pigs were purchased from Jiafeng Pig Breeding Co., Ltd. in Jining, Shandong Province. All animal experimental procedures were approved by the Ethics Committee of the Affiliated Hospital of Inner Mongolia Medical University (Approval Number: DW2025022—Fu 01) and were carried out in accordance with the “Guide for the Care and Use of Laboratory Animals” of the National Institutes of Health (NIH) of the USA. The Bama miniature pigs were placed in an SPF environment with a 12-h light/12-h dark cycle, a temperature range of 20–25 ℃, and free access to drinking water.

After adapting for one week, the animals were intramuscularly injected with Shutai (a mixture of hydrochloric atropine and hydrochloric zolazepam) at a dose of 15 mg/kg to establish a venous channel. After that, propofol was continuously injected intravenously (0.2 mg/kg/min) to maintain general anesthesia and loss of consciousness, and tracheal intubation was performed to maintain breathing (23). At the end of the surgery, 2% lidocaine was locally injected at the incision (or puncture site) to reduce postoperative pain. An artificial blood vessel was sutured at the aortic bifurcation of the female Bama miniature pigs to establish a cystic abdominal aortic aneurysm model. One week later, a covered stent was implanted from the aorta to the right iliac artery to isolate the aneurysm and rely on the left iliac artery reflux to establish the T2EL model. The animals were randomly assigned to the TCC group (*n* = 3) or the IDC control group (*n* = 3) using computer-generated sequences. Blinding was not applied during the intervention period, but it was employed in the histological analysis. After the embolization procedure, the animals were monitored for limb perfusion, activity level, appetite, and wound healing conditions. The determination of the sample size was based on previous literature studies (24) as well as practical considerations related to model complexity, animal welfare, and ethical principles aimed at minimizing the use of animals. No formal *a priori* power calculation was conducted.

Postoperatively, animals were monitored daily for limb perfusion, activity level, appetite, and wound healing. No signs of distal limb ischemia, organ dysfunction, or behavioral abnormalities were observed. The primary endpoint was complete occlusion of the aneurysm sac with absence of persistent T2EL on imaging at 4 weeks. Secondary endpoints included evidence of distal embolization, thrombus organization on histology, and procedural complications. Imaging follow-up was performed at 2 and 4 weeks post-operation using color Doppler ultrasound and CT angiography (CTA). These timepoints were selected to evaluate early thrombus stabilization and short-term durability in this preclinical feasibility model. While longer follow-up would be valuable to assess recanalization risk, the current study focused on early sac occlusion efficacy. After the experiment, referring to the AVMA Guidelines for the Euthanasia of Animals: 2020 Edition and previous studies (25), when the animals showed no eyelid reflex and no pain sensation reflex and remained under deep anesthesia, we induced cardiac arrest by intravenous injection of 10% potassium chloride solution and performed euthanasia. Subsequently, all the animal vascular tissues were collected for histological (H&E) and immunofluorescence (α-SMA) analysis.

### 2.10 Embolization procedure details

Due to the irregular shape of the artificial pouch, it is impossible to conduct quantitative measurements of the pouch volume or the thrombus volume. After modeling and confirmation through DSA angiography, the existence of T2EL was clearly identified. Details of the embolization operation: The number of spring coils used was 1, and the length was 60 mm.

The dosage of thrombin delivered for each aneurysm: The average drug loading dose for making TCC was 0.0066 ± 0.0003 (g).

Main complications: 1. Bleeding, including bleeding at the puncture site and abdominal bleeding, etc.; 2. Incision infection; 3. Complications such as type I and type III endoleak after intracavitary isolation treatment of covered stent.

### 2.11 Dynamic coagulation assay (*ex vivo* flow model)

The definition of the *in vitro* dynamic flow model and the occlusion time.

To evaluate the procoagulant effect of TCC under hemodynamic conditions, an *in vitro* flow model was established. Fresh whole blood was collected from the jugular vein puncture site of the Bama miniature pig and injected into a vacuum tube containing a 3.8% phosphate citrate solution (the ratio of blood to anticoagulant was 9:1) to prevent blood clotting. The collection was performed within 2 h. For the dynamic circulation experiment, the anticoagulated blood circulated through a closed circulation flow system at a constant flow rate of 80 milliliters per min at room temperature (23 °C). The coagulation process was initiated immediately before the start of the circulation by recalcification (adding calcium chloride to reverse citrate anticoagulation). The occlusion time was defined as the interval from the addition of calcium chloride (time zero) to the complete cessation of downstream flow, which was determined by the flow-free/outflow pressure drop section. All experiments were repeated three times.

### 2.12 Hematoxylin and eosin (HE) staining

After the experiment, tissue samples from the abdominal aortic aneurysm segments of each group were collected and fixed in 4% paraformaldehyde (Beyotime, Shanghai, China; P0099) for 24 h. Samples were dehydrated through a graded ethanol series, cleared in xylene, and embedded in paraffin. Sections approximately 4 μm thick were prepared using a rotary microtome (Leica RM2235, Germany), deparaffinized in xylene, and rehydrated through descending concentrations of ethanol. Standard hematoxylin-eosin staining was performed using a commercial HE staining kit (Servicebio, Wuhan, China; G1120) according to the manufacturer's instructions. After dehydration and mounting with neutral resin, sections were examined and photographed under a light microscope (Olympus BX53, Japan).

### 2.13 Transcriptomic analysis

RNA was extracted from the TCC embolized abdominal aortic aneurysm (AAA) tissues and the normal aortic tissues (near the abdominal aorta trunk). The “normal aorta” samples were taken from the non-aneurysmal segments of the same animals as the baseline reference for transcriptomics. The quality of total RNA was evaluated using the Agilent 5400 bioanalyzer. For each sample, 5 μL of RNA was used for integrity and quantity assessment. All samples met the library construction standards (passed), and the RNA quality values ranged from 4.4 to 5.8 (Supplementary Table 1). Differential expression analysis was performed using the DESeq2 software, and the Benjamini-Hochberg false discovery rate (FDR) correction was applied; genes with |log_2_FC| > 2.5 and a corrected *p*-value (padj) < 0.001 were considered to have significant differences.

### 2.14 Functional enrichment analysis

The researchers used the clusterProfiler software package of R language to conduct gene ontology (GO) annotation and Kyoto Encyclopedia of Genes and Genomes (KEGG) pathway enrichment analysis for the differences. The GO enrichment analysis covered three major categories: biological process (BP), molecular function (MF), and cellular component (CC). The KEGG analysis identified significantly enriched signaling pathways. The study adopted *p* < 0.05 and *q* < 0.05 as the significance thresholds to determine whether the enrichment degree reached statistical significance.

### 2.15 Protein-Protein interaction (PPI) network construction

The researchers imported the differential genes into the String database (https://string-db.org/), and set the minimum interaction confidence level to 0.4 to obtain the protein-protein interaction (PPI) data. Because the STRING database does not provide a comprehensive annotation for Bama minipigs, porcine genes were converted to their corresponding human orthologs prior to PPI analysis, and Homo sapiens was selected for network construction. The PPI network was visualized using the Cytoscape software, and the network topology was analyzed by combining the cytoHubba plugin with the Maximum Clique Centerality (MCC) algorithm to select the top 10 hub genes.

### 2.16 Quantitative real-time PCR (qRT-PCR)

To measure the transcriptional levels of key genes, total RNA was extracted from the thrombotic abdominal aortic aneurysm tissues (with 3 animals in each group) and the adjacent normal abdominal aortic tissues of the same batch of animals using Trizol reagent (Invitrogen, USA) in accordance with the manufacturer's instructions. RNA purity and concentration were assessed with a NanoDrop 2000 (Thermo Fisher Scientific, USA). Complementary DNA (cDNA) was synthesized and amplified using the UniPeak U + One Step RT-qPCR SYBR Green Kit (Novoprotein, China) on a QuantStudio™ 6 Flex real-time PCR system (Thermo Fisher). The cycling program included reverse transcription at 55 °C for 10 min, pre-denaturation at 95 °C for 1 min, followed by 40 cycles of 95 °C for 15 s and 60 °C for 60 s, and melting-curve analysis. Relative gene expression levels were calculated using the 2^^−ΔΔCt^ method, with GAPDH and *β*-actin as internal controls, and all reactions were performed in triplicate. The primer sequences are shown in Table 1.

**Table 1:** The primer sequences.

Gene	Forward primer (5′→3′)	Reverse primer (5′→3′)
VDR	AATGGCGGCCAGCACTTCCC	CTGGCAGTGGCGTCGGTTGT
MMP9	ACCGGCTCTAAAGCTTCTCC	TCAAAGGTCTGGAATTTGCC
SLC2A5	CGGCTCCTCCTTCCAGTATG	GGACACAGACACAGACCACA
MKI67	AGTCTGTAAGGAAAGCCACCC	ACAAAGCCCAAGCAGACAGG
RUNX2	CAACTTCCTGTGCTCTGTGCT	GAGAACCAGGGTTGAGGTGAT
BTG2	TGGTTTCCTGAAAAGCCATC	GGACACTTCATAGGGGTCCA
PMP22	CTCCACGATCGTCAGCCAAT	GTGAAGAGCTGGCAGAAGAACAG
IGFBP6	CCCTCGGGGGAGAATCCTAA	GAGGGAGTGGTAGAGGTCCC
LGR5	GCCTTTGTAGGCAACCCTTC	AGGCACCATTCAAAGTCAGTG
LEPR	GAAAAACACCGGAATGATGC	AAAAGAAGAGGGCCAAATGTC
GAPDH	ACACTCACTCTTCTACCTTTG	CAAATTCATTGTCGTACCAG
*β*-actin	AGTTGAAGGTGGTCTCGTGG	TGCGGGACATCAAGGAGAAG
MMP7	GAATGACTGGATTGTGGCTC	GGGCGACTTTCCTTTAGC

### 2.17 Residual solvent control

After the coating treatment, all TCC equipment was continuously dried at room temperature in a well-ventilated fume hood for 24 h to ensure that the residual dichloromethane was completely evaporated. The rapid evaporation property of dichloromethane and the small volume of solvents used during the coating process can minimize the possibility of residual solvents remaining.

### 2.18 Sterilization procedure

After the coating dries, it undergoes low-temperature disinfection and is stored in a sterile condition. All TCC devices were sterilized using hydrogen peroxide plasma before being used *in vivo* (26, 27). The sterilization process followed the manufacturer's specified requirements and standard parameters (temperature, pressure, and duration). Neither CT angiography nor histological examination revealed any signs of distal thrombosis or organ infarction.

### 2.19 Statistical analysis

All statistical analyses were performed using GraphPad Prism 9 (GraphPad Software, USA) and R version 4.1.0 (Austria). Data are presented as mean ± standard deviation (SD). Comparisons between two groups were analyzed using unpaired two-tailed Student's *t*-test. Multiple group comparisons were analyzed by one-way ANOVA followed by Tukey's *post hoc* test. A two-sided *p* < 0.05 was considered statistically significant.

## 3 Results

### 3.1 TCC fabrication and basic properties

**Figure 1:**
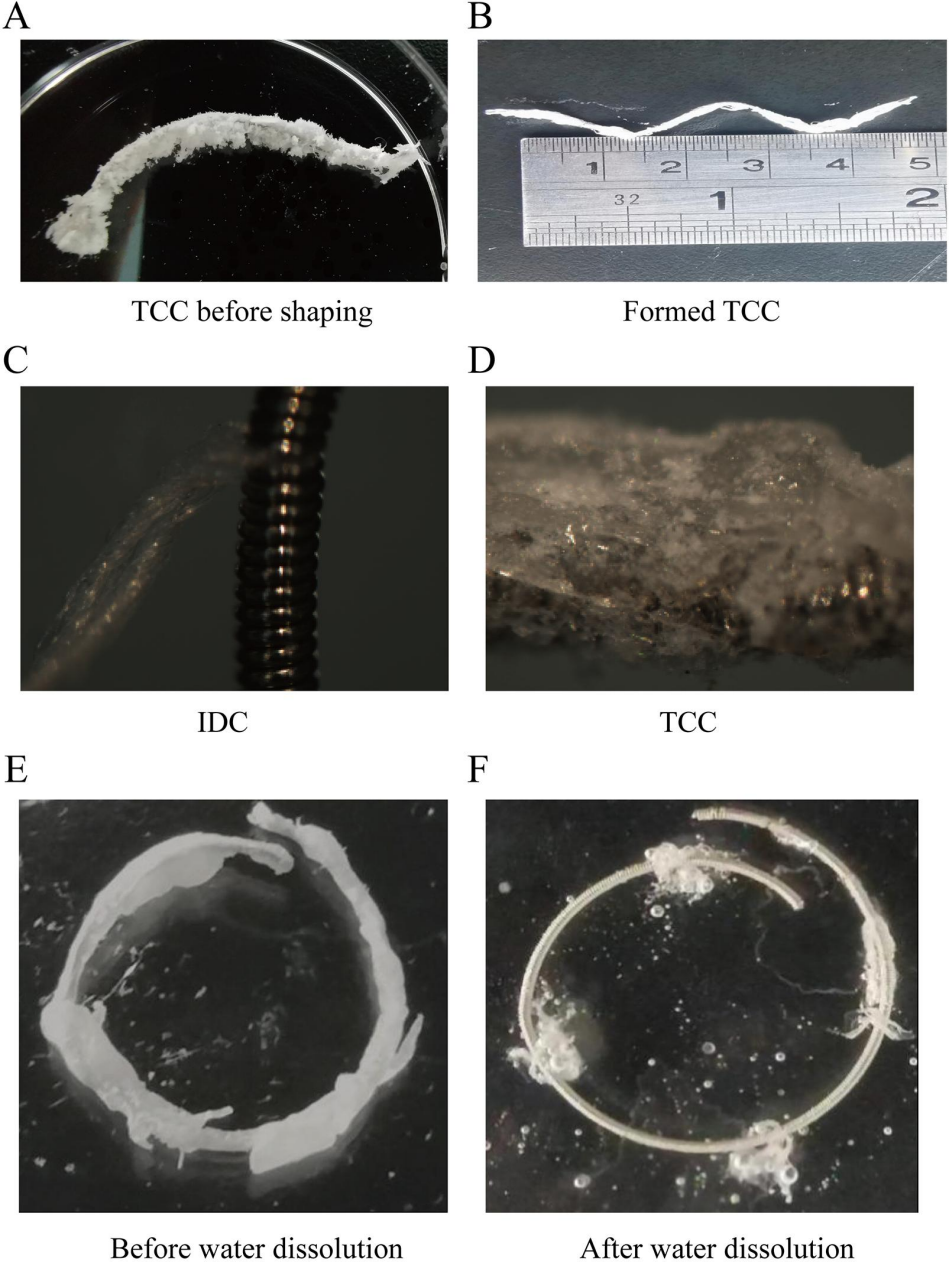
TCC fabrication and basic properties. **(A)** TCC before shaping. **(B)** Formed TCC. **(C,D)** Observation of the LTP coating adhesion of IDC and TCC under an 8× magnification microscope. **(E,F)** TCC before water dissolution and after water dissolution.

The TCC was successfully fabricated with a uniform, firm coating of LTP (Figures 1A,B). Using an optical microscope to observe the structure of TCC and the adhesion of the surface crystalline coating, the results showed that the LTP coating structure successfully adhered to the surfaces of IDC and TCC (Figures 1C,D). The average quality of TCC was 0.0066 ± 0.0003 g. The PVP/PCL coating provided a controlled dissolution time of 1,080 ± 42.43 s, which was optimized to be longer than the initiation of coagulation (Table 2 and Figures 1E,F).

**Table 2:** Water-dissolution time of PVP and PCL media of different proportions.

PVP(g)	PCL(g)	Water-dissolution time(s)
0.7	0.5	2,598.33 ± 79.1
0.9	0.3	1,471.67 ± 77.05
1.1	0.1	1,080.00 ± 42.43
1.05	0.05	711.67 ± 35.45
1.07	0.03	246.67 ± 54.28

### 3.2 Superior coagulation performance of TCC

**Figure 2:**
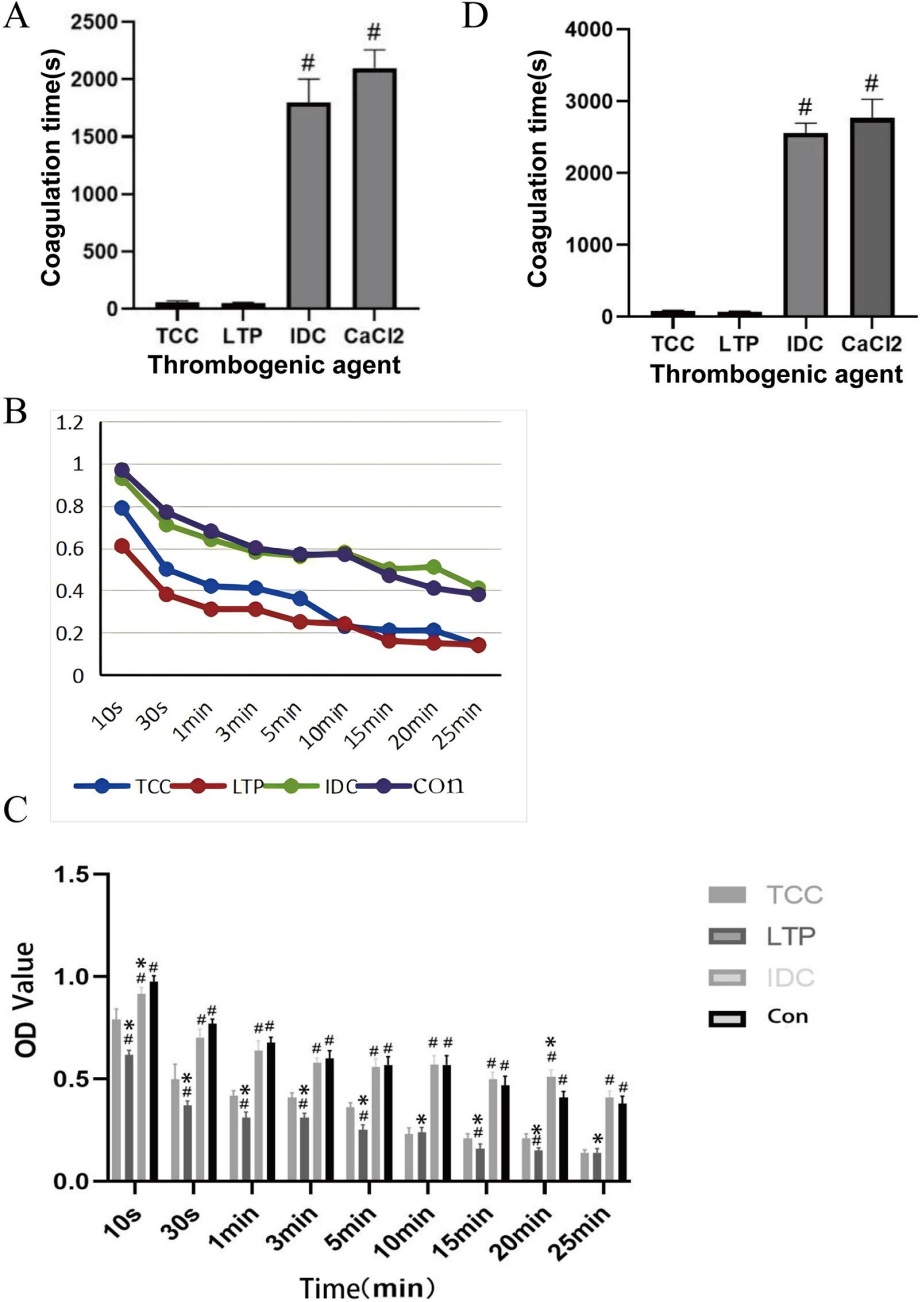
Coagulation performance of TCC. **(A)** Static coagulation test—coagulation time analysis graph. **(B)** The curve of OD value change in the static coagulation experiment. **(C)** Analysis Chart of OD Value Changes in Static Coagulation Experiment. **(D)** *ex vivo* dynamic coagulation experiment analysis. #*p* < 0.05 vs. TCC, *p < 0.05 vs. Control.

In the static coagulation assay, TCC and LTP dramatically shortened coagulation time compared to IDC and the control (*p* < 0.05) (Figure 2A). The OD value analysis confirmed the rapid coagulation kinetics of TCC and LTP (*p* < 0.05) (Figures 2B,C). In the dynamic flow model, The time taken for TCC to achieve occlusion was comparable to that of the LTP group, and was significantly faster than that of the IDC group and the control group (*p* < 0.05) (Figure 2D). Notably, free LTP caused ectopic embolism in the outflow circuit, a phenomenon not observed with TCC.

### 3.3 Biological effects of TCC on vascular CellsTCC

**Figure 3:**
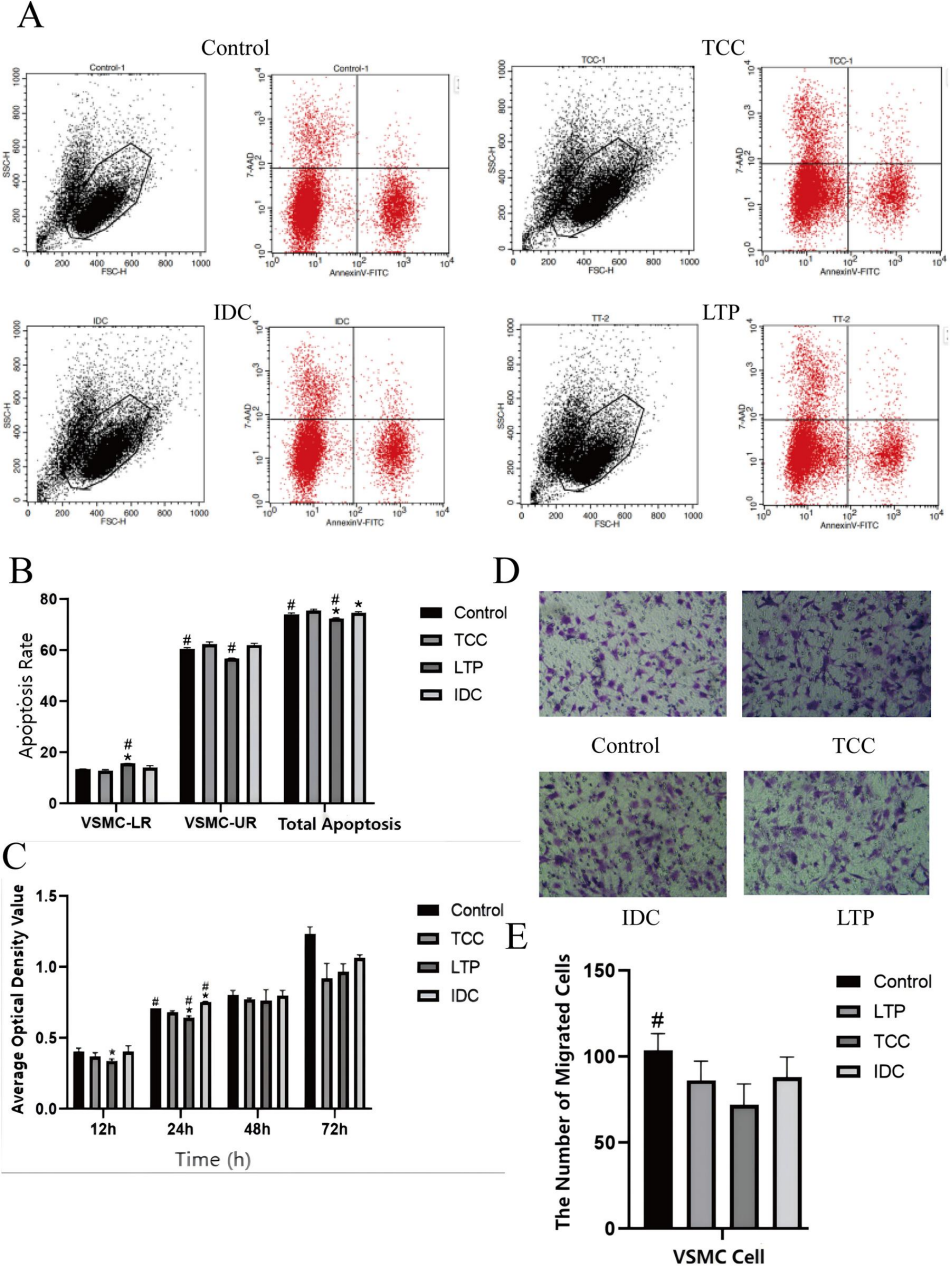
The effect of TCC on the biological function of vascular smooth muscle cells (VSMCs). **(A)** Apoptosis experiment. **(B)** Statistical chart of apoptosis experiment data. **(C)** CCK-8 experiment determines cell viability. **(D)** Migration experiment. **(E)** Migration Capability Analysis. #*p* < 0.05 vs. TCC, *p < 0.05 vs. Control.

**Figure 4:**
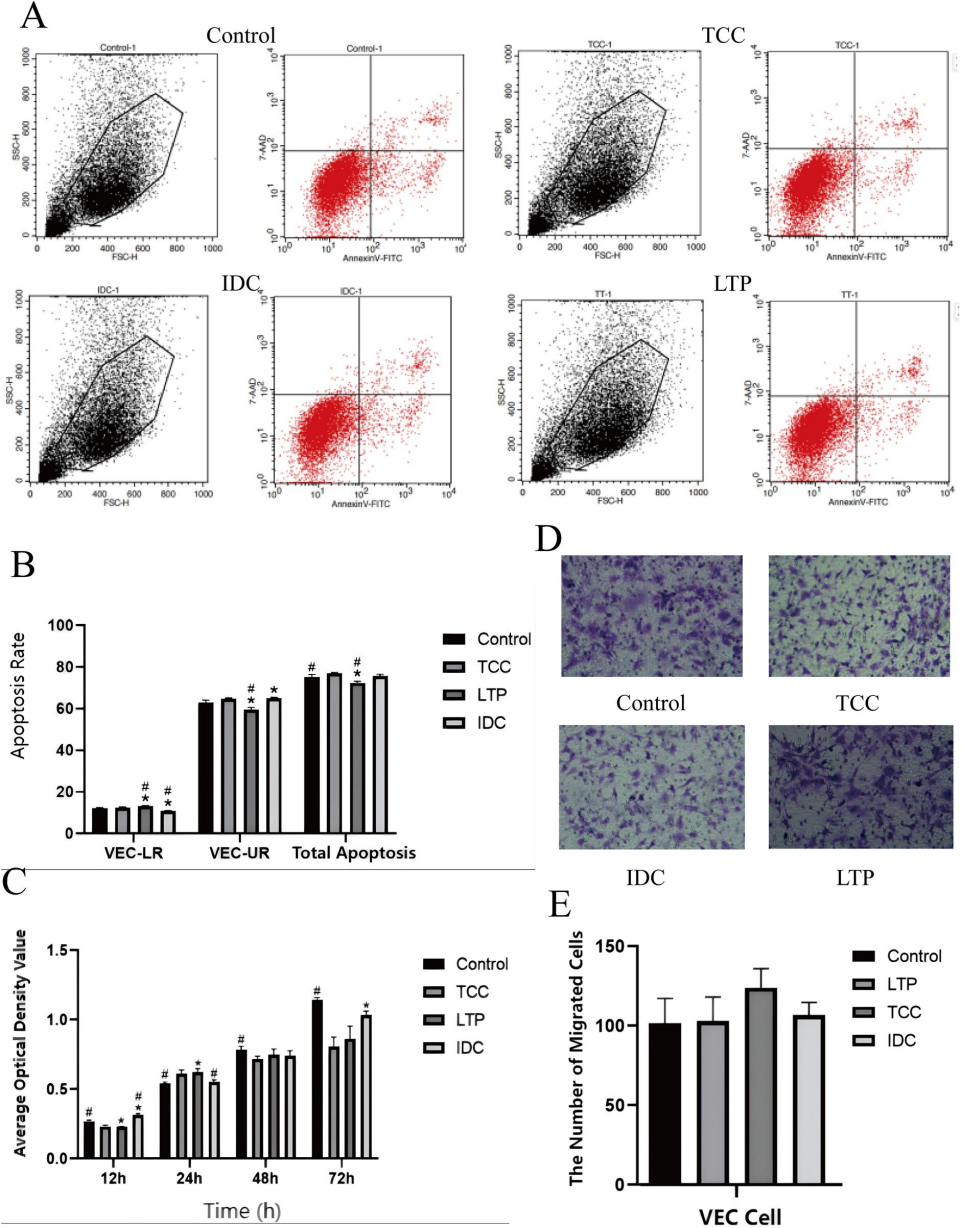
The effect of TCC on the biological function of vascular smooth muscle cells (VECs). **(A)** Apoptosis experiment. **(B)** Statistical chart of apoptosis experiment data. **(C)** CCK-8 experiment determines cell viability. **(D)** Migration experiment. **(E)** Migration Capability Analysis. #*p* < 0.05 vs. TCC, *p < 0.05 vs. Control.

**Figure 5:**
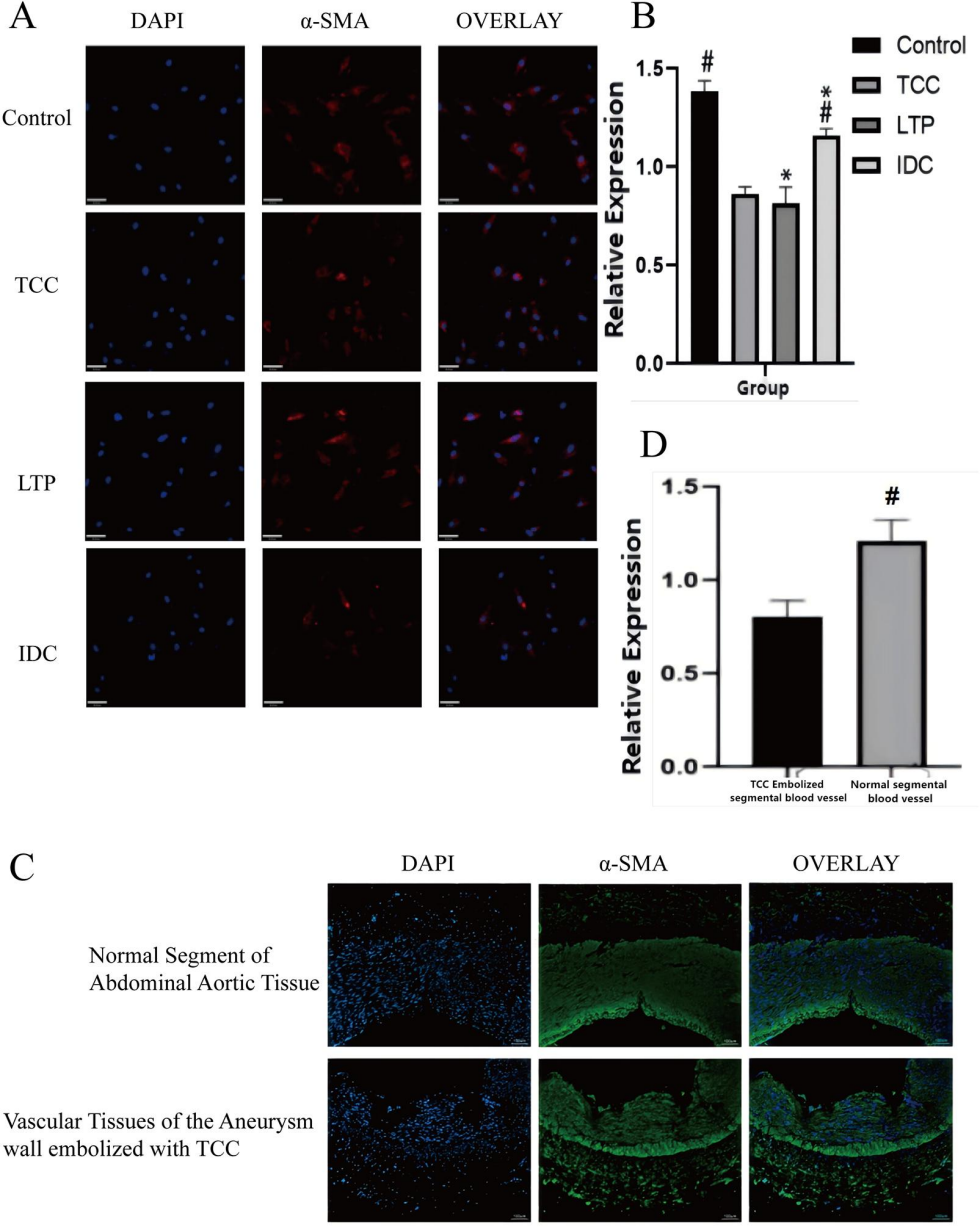
The effect of TCC on α-SMA *in vitro* and *in vivo*. **(A)** Fluorescence Map of a-SMA Expression in VSMC cells, with A Scale of 80um. **(B)** α-SMA relative expression analysis of VSMC cells. **(C)** Immunofluorescence detection of α-SMA expression in normal abdominal aortic tissue and TCC embolized abdominal aortic. **(D)** Relative expression analysis of α-SMA *in vivo*. #*p* < 0.05 vs. TCC, *p < 0.05 vs. Control.

Since successful prevention of T2EL primarily depends on rapid and stable thrombus formation within the aneurysmal sac, we further investigated the biological responses of vascular cells to TCC. TCC treatment significantly induced late and total apoptosis in VSMCs compared to the control and reduced their proliferation at 24 h and migration capacity (all *p* < 0.05) (Figures 3A–E). For VECs, TCC increased total apoptosis (*p* < 0.05) (Figures 4A,B) and showed a complex time-dependent effect on proliferation (*p* < 0.05) (Figure 4C), but did not significantly affect migration (*p* > 0.05) (Figures 4D,E). Furthermore, *in vitro* experiments demonstrated that TCC significantly downregulated the expression of the contraction marker α-SMA in vascular smooth muscle cells (VSMCs) (*p* < 0.05) (Figures 5A,B). Consistent with the results from the *in vitro* experiments, the *in vivo* experiments showed that the expression level of α-SMA in the vascular walls affected by TCC embolism was significantly reduced (*p* < 0.05) (Figures 5C,D).

### 3.4 *In vivo* efficacy in a minipig T2EL model

**Figure 6:**
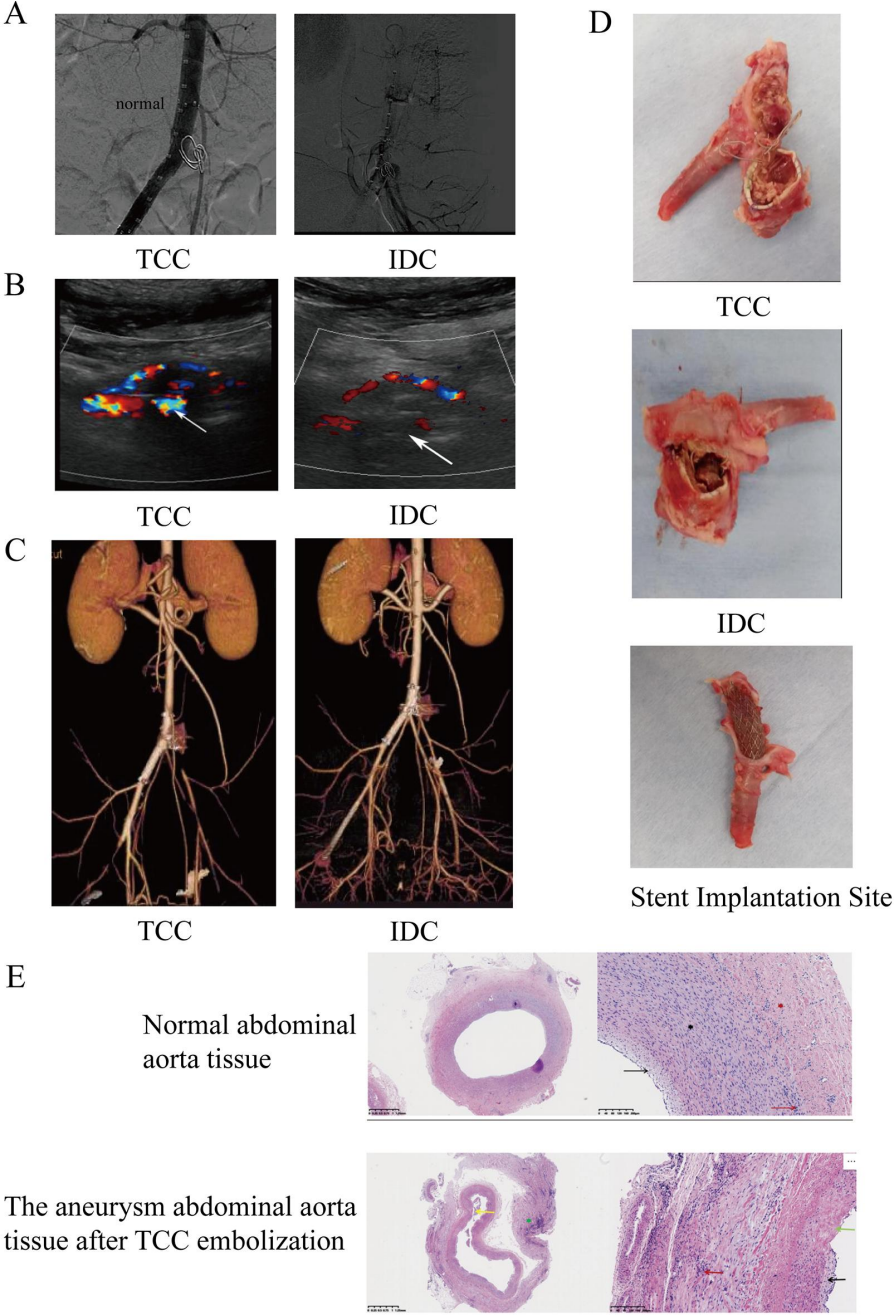
The influence of TCC on the T2EL model. **(A)** Schematic diagram of TCC and IDC embolization treatment. **(B)** Color Doppler Reexamination Results. **(C)** Postoperative Abdominal Aortic CTA. **(D)** Organism specimen testing. E: HE staining for detecting pathological changes.

AAA and T2EL models were successfully established in all animals. Embolic treatment using TCC and IDC was performed (Figure 6A). During the follow-up period, no signs of distal limb ischemia, organ dysfunction, or behavioral abnormalities were observed in treated animals. CTA imaging did not reveal evidence of distal embolization or organ infarction. Color Doppler examination two weeks after embolization and CT angiography (CTA) four weeks after surgery showed that the aneurysm cavity was completely and persistently occluded after TCC embolization, forming a thrombus. In the IDC group, blood flow could still be observed within the aneurysm cavity (Figures 6B,C). Tissue specimen detection revealed that structured thrombus formation was observed in the aneurysm cavity of the TCC group, while only a small amount of thrombus remained in the IDC group. The implanted covered stent was intact, adhered closely to the vascular endothelium, and did not shift (Figure 6D). H&E staining showed that compared with the normal abdominal aorta, there were significant thickening of the intima, shedding of vascular wall endothelial cells and necrosis, a decrease in the number of cells in the media layer, disordered arrangement, thickening of the adventitia layer, and inflammatory cell infiltration in the abdominal aortic aneurysm tissue after TCC embolization (Figure 6E).

### 3.5 Transcriptomic analysis

**Figure 7:**
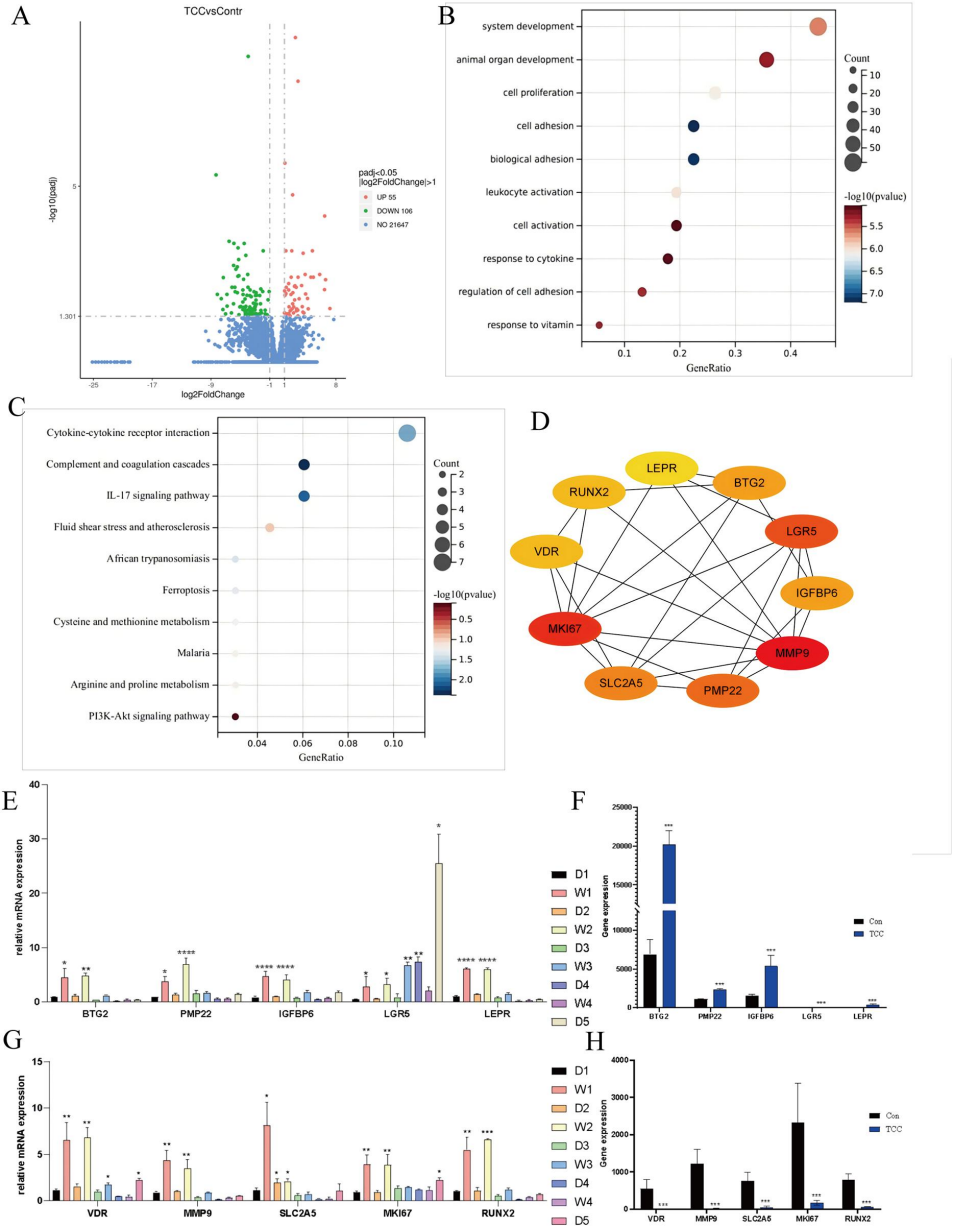
Transcriptomic analysis. **(A)** Differential gene volcano plot. **(B)** GO enrichment analysis. **(C)** KEGG Pathway Enrichment Analysis. **(E)** Verification of the expression of upregulated genes by qRT-PCR. **(F)** Transcriptome upregulated gene data. **(G)** Verification of the expression of downregulated genes by qRT-PCR. **(H)** Transcriptome downregulated gene data.

To investigate molecular changes in the vascular wall following TCC embolization, we compared the transcriptomic profiles of TCC-embolized aneurysm tissue and normal aortic tissue from the same animals. A total of six RNA sequencing libraries were constructed and sequenced. After adapter trimming and low-quality filtering, 124,123,239 (TCC) and 124,122,399 (control) clean reads were retained. The sequencing output per sample ranged from 5.83 to 6.47 Gb. The overall Q20 and Q30 scores were 97.28% and 93.00% respectively (Supplementary Table 2). The results showed that there were 161 differentially expressed genes between TCC embolization group and the normal aortic tissue, among which 55 were upregulated and 106 were downregulated (Figure 7A). GO enrichment analysis indicated that these differentially expressed genes were mainly involved in processes such as system development, cell proliferation, and cell adhesion (Figure 7B). KEGG analysis revealed that these differentially expressed genes were mainly involved in “cytokine-cytokine receptor interaction”, “complement and coagulation cascade reaction”, and “IL-17 signaling pathway” (Figure 7C). The protein-protein interaction analysis of the genes obtained from the transcriptome analysis was performed using the STRING database, combined with the results of animal pathology detection, and key genes were screened out. Subsequently, the CytoHubba plugin in Cytoscape software was used to screen out the key genes (Figure 7D). Subsequently, qRT-PCR validation confirmed the differential expression of selected genes: BTG2, PMP22, IGFBP6, LGR5, and DPT were significantly upregulated, while MMP9, VDR, E2F8, MKI67, and RUNX2 were significantly downregulated in TCC-embolized tissue compared to normal aorta (Figures 7E–H), consistent with the RNA-seq results.

## 4 Discussion

This study comprehensively evaluated the feasibility of a new embolic material—thrombin-coated controllable coils-in preventing T2EL after EVAR through systematic *in vitro* experiments, cytological assessment, animal model validation, and transcriptomic analysis.

The primary goal of T2EL prevention is to achieve rapid and sustained thrombosis occlusion within the aneurysm sac. The successful preparation of TCC relies on the stable loading of thrombin by the PVP/PCL coating system. PVP, as a water-soluble, biocompatible and safe polymer material, has been approved by the US FDA as a GRAS excipient for use in the pharmaceutical and food industries, and is widely applied in cardiovascular biomaterials and controlled-release systems (28–30). PCL, a biodegradable and biocompatible aliphatic polyester, is commonly applied in medical devices, drug delivery, and tissue engineering (31, 32). In this study, PVP and PCL were dissolved in dichloromethane at different ratios. The rapid volatility of dichloromethane, the adhesion property of PVP and the controlled degradation release and water solubility time of PCL were utilized to ensure that LTP was firmly adhered to the surface of IDC. Optical microscopy confirmed that LTP was successfully attached to the surface of IDC, with a uniform coating structure and strong adhesion. By adjusting the ratio of PVP/PCL, a controllable water dissolution time (1,080 ± 42.43 s) could be obtained. This time was slightly longer than the thrombin activation time, which not only ensured the release of thrombin at the target site but also avoided its early detachment and the occurrence of ectopic embolism.

The static coagulation experiment showed that the coagulation time in the TCC and LTP groups was significantly shorter than that in the IDC group and the control group. The curve of OD value changes also confirmed that TCC had rapid coagulation kinetics characteristics. In the *in vitro* dynamic circulation model, the complete occlusion time of TCC was comparable to that of the LTP group, and was significantly faster than that of the IDC group and the control group. It is notable that the free LTP group observed ectopic embolism in the outflow tract, while the TCC group did not, indicating that the PVP/PCL coating can effectively anchor thrombin on the surface of the IDC, preventing it from drifting with the blood flow to non-target sites. These findings indicate that localized thrombin release enables rapid sac thrombosis, representing the primary mechanism for T2EL prevention. To simulate the real blood flow environment, this study used 3D printing technology to construct *in vitro* models of AAA and T2EL, and combined with quartz transparent channels to observe the process of thrombus formation in real time. Although this model has certain differences in vascular elasticity and hemodynamics compared to human blood vessels, it still provides a repeatable and visualized effective platform for evaluating the thrombus formation ability of TCC under blood flow conditions (33, 34).

Successful T2EL prevention not only depends on rapid thrombosis, but also needs to take into account the biological effects of the embolic material on the cells of the vascular wall. Atherosclerosis is one of the main causes of AAA formation (35). Endothelial dysfunction promotes atherosclerosis (36), while endothelial migration is essential for vascular repair and remodeling (37). In atherosclerotic progression, disordered lipid metabolism induces phenotypic switching of VSMCs from contractile to synthetic states (38, 39), thereby enhancing proliferation and migration (40, 41). The results of this study indicate that TCC treatment promotes the apoptosis of vascular smooth muscle cells (VSMCs), while reducing their proliferation, migration, and α-SMA expression levels. This suggests that the contractile phenotype is inhibited and excessive vascular remodeling can be prevented. These results suggest that TCC may regulate the biological functions of VSMCs. The *in vivo* experiments further confirmed that the expression level of α-SMA in the vascular wall significantly decreased after TCC embolization. However, the downregulation of α-SMA may occur through two mechanisms: either VSMCs transform from a contractile type to a synthetic type, or VSMCs undergo apoptosis resulting in a reduction in cell number. In this study, *in vitro* TCC treatment significantly increased VSMC apoptosis and decreased cell viability, suggesting that cell loss is one of the reasons for the weakened α-SMA signal; the H&E staining in the *in vivo* experiment also showed a reduction in the number of medial cells in the TCC embolized area, which is consistent with the cell loss. However, whether the surviving VSMCs undergo phenotypic transformation still needs further verification. Therefore, although TCC regulates the biological characteristics of VSMCs, whether the downregulation of α-SMA represents “favorable remodeling” is not clear and requires further in-depth analysis in combination with more phenotypic markers.

In this study, a cystic aneurysmal bulge (AAA) model was constructed at the aortic bifurcation of the Bama miniature pigs through surgical procedures, and a T2EL model with left iliac artery reflux as the source was established. This model has good anatomical stability and hemodynamic similarity, providing a reproducible preclinical platform for T2EL embolization research (42–46). The results showed that neither the animals in the TCC group nor those in the IDC group exhibited limb ischemia, organ dysfunction, or behavioral abnormalities. No distant embolism or organ infarction signs were observed in the CTA examination. Color Doppler ultrasound at 2 weeks postoperatively and 4 weeks of CTA examination revealed that the aneurysm sac in the TCC group was completely occluded, with stable and continuous thrombus formation; while in the IDC group, blood flow signals could still be seen in the aneurysm sac. H&E staining showed that after TCC embolization, the AAA tissue showed significant thickening of the intima, and necrosis of endothelial cells, reduction and disordered arrangement of the media cells, thickening of the adventitia, and infiltration of inflammatory cells, suggesting that the local tissue had an adaptive response to the embolic material.

To deeply elucidate the molecular mechanism underlying the vascular changes induced by TCC, this study conducted transcriptome sequencing analysis on AAA tissues treated with TCC embolization and the normal aortic tissues from the same animal. GO enrichment analysis revealed that the differentially expressed genes were mainly involved in biological processes such as development, cell proliferation, and cell adhesion. KEGG pathway enrichment analysis indicated that the differentially expressed genes were significantly enriched in “cytokine-cytokine receptor interaction”, “complement and coagulation cascade reaction”, and “IL-17 signaling pathway”. BTG2, PMP22, IGFBP6, LGR5 and DPT were significantly upregulated, while MMP9, VDR, E2F8, MKI67 and RUNX2 were significantly downregulated. These results suggest that TCC may inhibit matrix degradation and cell proliferation.

MMP is a type of zinc dependent endopeptidase protein (47) playing a role in pathological diseases related to physiological processes such as angiogenesis. The matrix metalloproteinases produced by VSMC can cause degradation of elastin and collagen, which is closely related to the occurrence of AAA. MMP-2 and MMP-9 are the main proteases that degrade elastic fibers in AAA, leading to the formation and development of AAA (48). In this study, compared with healthy aortic tissue, MMP-9 and MMP-7 levels were downregulated in arterial aneurysm aortic specimens after TCC embolization, indicating that TCC embolization material may have inhibitory effects on AAA proliferation, migration, and other functions, which is consistent with the results of *in vitro* cytology experiments.

Although this study systematically evaluated the performance of TCC from multiple aspects such as materials science, cell biology, animal models, and transcriptomics, there are still some limitations that need to be improved in subsequent research. In terms of materials and preparation processes, first, the coating thickness was not quantitatively measured (such as SEM cross-sectional analysis), which limited the precise characterization of the coating structure. Second, the uniformity of the coating was only observed through optical microscopy and not quantitatively evaluated using high-resolution techniques (such as SEM). Third, although dichloromethane has high volatility and the coating was fully dried after application, quantitative residual solvent analysis was not conducted. In the future, gas chromatography should be used to detect residual solvents in accordance with relevant standards to ensure compliance with safety thresholds. Fourth, although the load mass of thrombin and the water dissolution time were measured, quantitative detection of thrombin activity (such as the colorimetric substrate method) and release kinetics were not performed. Future research should supplement relevant experiments to clarify the retention rate and release behavior of thrombin on the coating. Fifth, thrombin activity was not quantified in international units (IU), nor was the impact of the coating and sterilization treatment on thrombin activity evaluated. The existing functional experiments confirmed its procoagulant activity, but direct quantitative enzyme activity measurement can provide a more rigorous standardized basis. In terms of VSMCs phenotype characterization, this study only detected one marker, α-SMA, and failed to systematically evaluate the changes in contraction-type (such as SM22α, calponin) and synthesis-type (such as osteopontin, vimentin) markers. Therefore, it is difficult to clearly distinguish whether VSMCs have undergone phenotypic transformation or a decrease in cell number. Future studies should adopt multiple immunohistochemistry or single-cell RNA sequencing techniques to analyze these mechanisms at a higher resolution. In terms of *in vivo* research, first, the sample size of this study was small, and no formal efficacy analysis was conducted, so the results should be regarded as preliminary explorations. Second, due to the irregular shape of the surgically constructed cystic AAA, it is impossible to quantitatively measure the aneurysm sac volume or thrombus volume. Third, although the AAA model constructed by surgery has good reproducibility, its vascular wall is composed of artificial vascular patches, lacking the degenerative extracellular matrix remodeling, inflammation, and adhesion thrombus characteristics of human AAA, and its biomechanical properties and hemodynamic characteristics differ from those in clinical practice. Future studies should employ models that are closer to the pathological environment (such as elastase-induced or xenogeneic abdominal aortic aneurysm models) to evaluate long-term performance and vascular remodeling. Fourth, the follow-up time was only 4 weeks, which is sufficient to evaluate the early embolization effect, but not sufficient to assess long-term durability, reperfusion risk, or long-term adverse events (such as coil compression, wall degeneration, etc.). Fifth, although this study did not detect distal embolization or organ infarction through CTA and gross observation, it did not systematically detect systemic coagulation parameters (such as PT, APTT, D-dimer), nor did it perform pathological examinations of distant organs, limiting the comprehensive assessment of systemic thrombus risk and subclinical coagulation abnormalities. Sixth, the formation of T2EL is influenced by multiple factors such as branch vascular anatomy, hemodynamics, coagulation status, and device characteristics. Although this study evaluated multiple materials and biological variables, the main determinant of aneurysm sac occlusion is still the rapid and stable thrombus formation. Therefore, the results of histological and transcriptomic observations should be regarded as exploratory findings rather than direct causal evidence for T2EL prevention. Seventh, the observed endothelial injury, reduction of the media cells, and inflammatory infiltration are both the expected local tissue response to foreign substances after embolization and may represent a certain degree of tissue damage. Long-term follow-up and detection of more markers (such as apoptosis detection, VSMCs phenotype markers, collagen deposition, etc.) will help clarify whether these changes are adaptive remodeling or damaging alterations. In terms of transcriptomics analysis, first, the comparison subjects of this study were AAA tissues with TCC embolization and normal aortic tissues from different anatomical locations. This design was unable to distinguish the pathological changes of AAA itself, the influence of the embolization operation, and the specific effects of TCC. The absence of AAA tissues without IDC embolization as a control limited the possibility of attributing molecular changes to the thrombin coating. Future studies should include untreated AAA tissues and IDC embolized tissues as controls to separate the TCC-specific molecular characteristics. Secondly, due to database limitations, when performing PPI analysis, pig genes were mapped to human homologous genes, which may introduce annotation bias or loss of species-specific interaction information. In the future, direct protein interaction studies should be conducted in pig tissues to verify this. Thirdly, the transcriptome data only represent a single time point (4 weeks after surgery) and cannot reflect the temporal dynamic changes in gene expression. The main value of these transcriptome data lies in providing hypotheses for subsequent mechanism studies. Future longitudinal studies at multiple time points are needed to reveal the time course of vascular remodeling.

## 5 Conclusion

In conclusion, this study successfully developed a new type of composite embolic material, TCC, which has a simple and practical preparation process, good thrombin loading capacity, controllable release characteristics, and efficient coagulation-promoting effects. Although this study has some limitations such as small sample size, model limitations, short follow-up time, and missing some safety indicators, overall, TCC, as a new generation of embolic material with controllability, efficient coagulation-promoting properties, and good biocompatibility, shows a promising application prospect in preventing T2EL after EVAR. Future research should be conducted in more clinical-like pathological models for long-term verification and improvement of systematic safety assessment to lay the foundation for its clinical translation.

## Data Availability

The original contributions presented in the study are included in the article/Supplementary Material, further inquiries can be directed to the corresponding authors.
