# Supplementary material for: Research and development of novel embolization materials and study on their feasibility of preventing T2EL after EVAR abdominal aortic aneurysm
Source: Front Cardiovasc Med. 2026 Jun 1;13:1740816. doi: 10.3389/fcvm.2026.1740816 (PMC13265520; doi:10.3389/fcvm.2026.1740816)
Supplement: Supplementary file 1 [file Datasheet1.docx]

|  | X2 | X4 | X6 | X8 |
| --- | --- | --- | --- | --- |
| IDC | 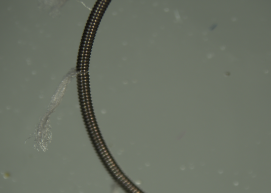 | 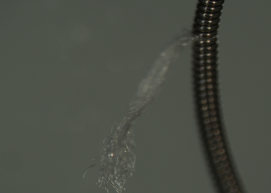 | 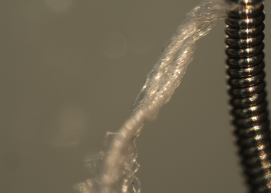 | 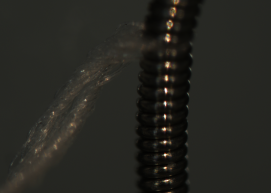 |
| TCC | 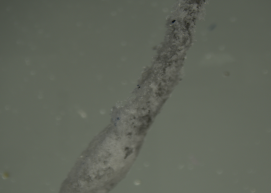 | 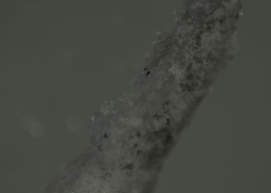 | 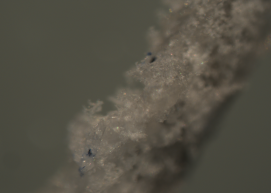 | 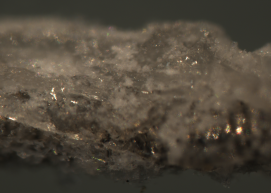 |

Supplementary Figure 1. Observation of TCC structural changes using an anatomical microscope.

| **Samples** | **Concentration(ng/ul)** | **Volume(ul)** | **Total quantity(ug)** | **Completeness value** | **Test conclusion** |
| --- | --- | --- | --- | --- | --- |
| 1 | 23 | 35 | 0.805 | 5.3 | Pass |
| 2 | 38 | 35 | 1.33 | 5.4 | Pass |
| 3 | 56 | 35 | 1.96 | 4.4 | Pass |
| 4 | 128 | 35 | 4.48 | 5.5 | Pass |
| 5 | 31 | 35 | 1.085 | 5.8 | Pass |
| 6 | 291 | 35 | 10.185 | 5.2 | Pass |

Supplementary Table 1. Detection results of RNA concentration and integrity.

Supplementary Table 2. Quality control and filtering results of transcriptome sequencing data.

| sample | raw_reads | raw_  bases | clean_  reads | clean_  bases | error_  rate | Q20 | Q30 | GC_pct |
| --- | --- | --- | --- | --- | --- | --- | --- | --- |
| TCC1 | 45730780 | 6.86G | 43105072 | 6.47G | 0.03 | 97.42 | 93.22 | 51.98 |
| TCC2 | 39948278 | 5.99G | 38869158 | 5.83G | 0.03 | 97.54 | 93.50 | 52.75 |
| TCC3 | 43635492 | 6.55G | 42265768 | 6.34G | 0.03 | 97.29 | 93.10 | 51.90 |
| Contr1 | 43479304 | 6.52G | 42132942 | 6.32G | 0.03 | 96.75 | 91.91 | 51.85 |
| Contr2 | 42463232 | 6.37G | 41155134 | 6.17G | 0.03 | 97.49 | 93.41 | 52.52 |
| Contr3 | 41327516 | 6.2G | 39950894 | 5.99G | 0.03 | 97.21 | 92.87 | 53.00 |
